# Supplementary material for: A cosmogenic 10Be anomaly during the late Miocene as independent time marker for marine archives
Source: Nat Commun. 2025 Feb 10;16:866. doi: 10.1038/s41467-024-55662-4 (PMC11811166; doi:10.1038/s41467-024-55662-4)
Supplement: Supplementary file 1 — Supplementary Information [file 41467_2024_55662_MOESM1_ESM.pdf]

Supplementary information to: A cosmogenic ^{10}Be anomaly during the late Miocene as independent time marker for marine archives

Dominik Koll[1,2,3*], Johannes Lachner[1], Sabrina Beutner[4], Sebastian Fichter[1], Silke Merchel[1,5], Georg Rugel[1], Zuzana Slavkovská[2], Carlos Vivo-Vilches[1,5], Stella Winkler[1], Anton Wallner[1,3]

[1]Accelerator Mass Spectrometry and Isotope Research, Helmholtz-Zentrum Dresden-Rossendorf, Dresden, 01328, Germany.
[2]Department of Nuclear Physics and Accelerator Applications, The Australian National University, Canberra, 2601, Australia.
[3]Institute of Nuclear and Particle Physics, TUD Dresden University of Technology, Dresden, 01069, Germany.
[4]Institute of Resource Ecology, Helmholtz-Zentrum Dresden-Rossendorf, Dresden, 01328, Germany.
[5]University of Vienna - Faculty of Physics, Vienna, 1090, Austria.

*Corresponding author(s). E-mail(s): d.koll@hzdr.de;

In this supplementary, the accelerator mass spectrometry measurement data for the crust VA13/2-237KD and SO142-4DR are displayed. The measurements were performed at the DREsden AMS (DREAMS) facility of Helmholtz-Zentrum Dresden-Rossendorf, Germany [1]. Uncertainties are calculated as 1-σ confidence levels and any error bars are smaller than the displayed data points. Furthermore, the stable element analysis of VA13/2-237KD by inductively-coupled plasma mass spectrometry and the normalisation of the ^{10}Be concentrations with respect to different matrix elements are given.

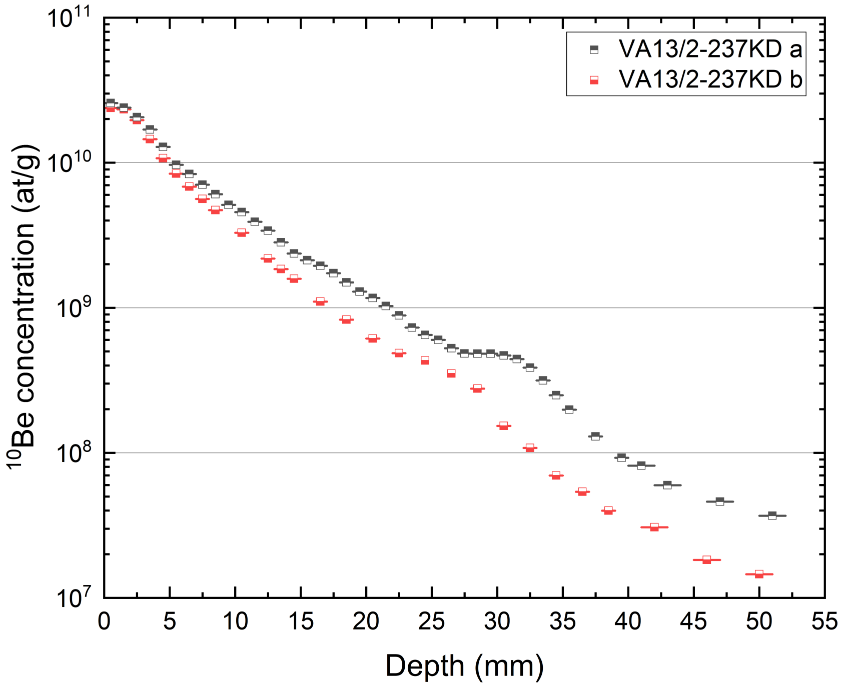

Supplementary Figure 1 ^{10}Be concentration vs. depth measured in crust VA13/2-237KD. The data indicate a different growth-rate of the crust depending on the lateral position of the drill-holes (a) and (b). An anomalous increase of ^{10}Be concentration can be seen at different depths of the crust.

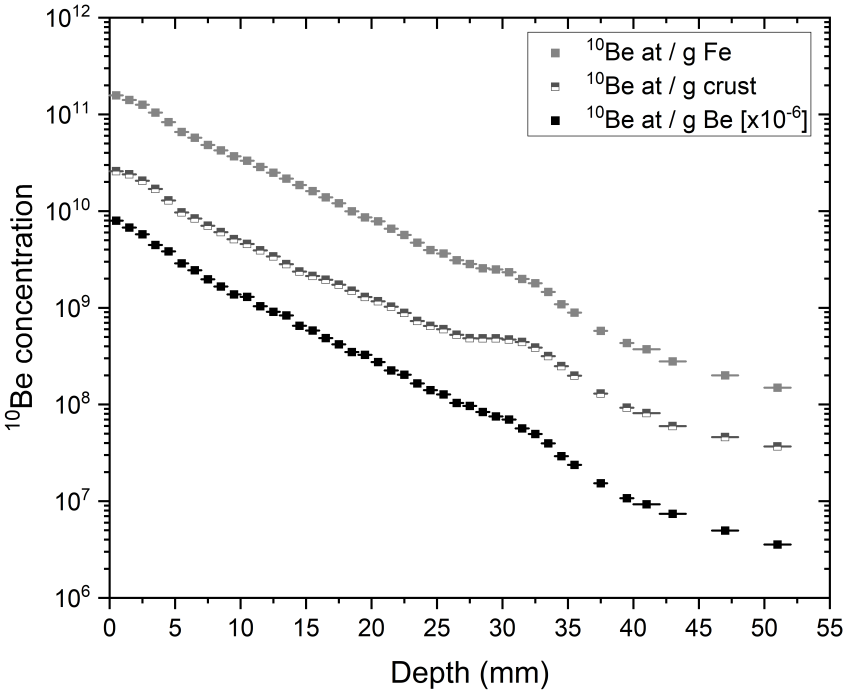

Supplementary Figure 2 ^{10}Be concentration vs. depth measured in crust VA13/2-237KD and normalized to different matrix elements. The canonical normalization to the mass of the ferromanganese crust samples (a measure for the Fe+Mn concentration) is displayed in comparison to a normalization to Be and to Fe concentrations. The ^{10}Be anomaly is present for all normalisations with varying amplitude, which points to a disconnected accumulation of trace ^{10}Be compared to minor stable Be or major stable Fe.

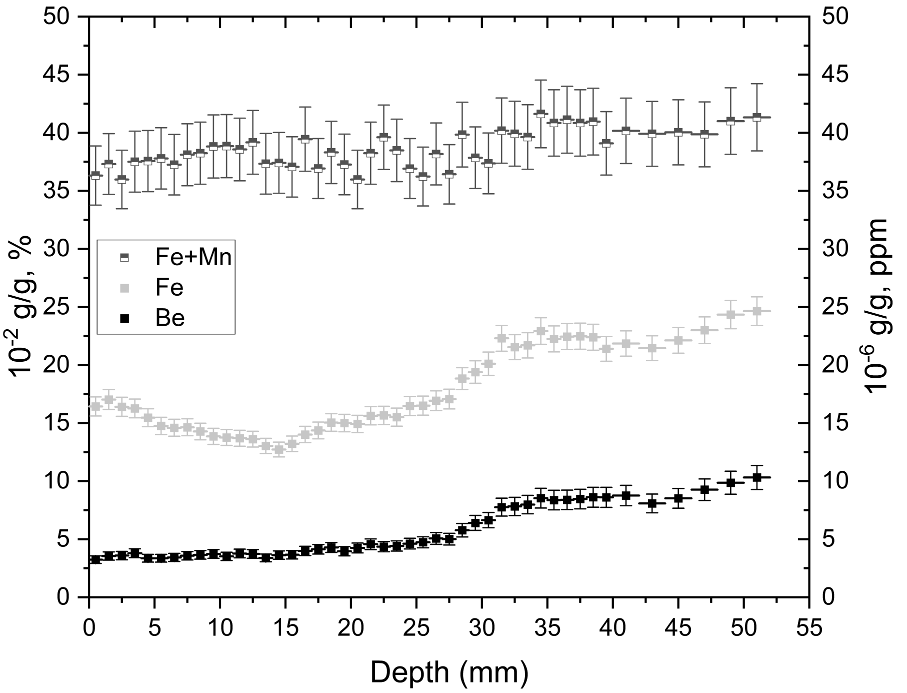

Supplementary Figure 3 Stable element concentrations of Be, Fe and Fe+Mn over depth in the ferromanganese crust VA13/2-237KD determined by inductively-coupled plasma mass spectrometry (ICP-MS). The stable Be concentration (right axis) increases monotonically with depth, in contrast to the ^{10}Be concentration. The total concentration of the main two matrix elements Fe+Mn (left axis) stays rather constant as expected from the genesis model of hydrogenetic ferromanganese crusts [2, 3]. The Fe concentration (left axis) is anti-correlated to the Be concentration for younger ages, whereas it is correlated for older ages implying a complex genesis with changing chemical or physical conditions or trace element concentrations in seawater.

	**Depth** mm	**Crust mass** mg	^{9}**Be carrier** μg	^{10}**Be/**^{9}**Be** 10^{-13} at/at	^{10}**Be** 10^{7} at/g
(a)	00 – 01	192	499	1485.4 ± 2.2	2580 ± 26
	01 – 02	194	502	1386.9 ± 3.2	2398 ± 25
	02 – 03	193	502	1186.6 ± 2.8	2062 ± 21
	03 – 04	191	502	964.8 ± 2.4	1694 ± 17
	04 – 05	192	503	735.1 ± 1.5	1287 ± 13
	05 – 06	193	502	557.2 ± 1.6	969 ± 10
	06 – 07	191	502	476.4 ± 1.4	837 ± 9
	07 – 08	193	501	407.1 ± 1.5	706 ± 7
	08 – 09	193	482	363.0 ± 1.1	607 ± 6
	09 – 10	193	503	293.8 ± 0.9	512 ± 5
	10 – 11	193	500	263.7 ± 0.9	457 ± 5
	11 – 12	192	501	224.8 ± 0.9	392 ± 4
	12 – 13	192	492	198.4 ± 0.9	339.7 ± 3.7
	13 – 14	190	501	160.4 ± 0.8	282.7 ± 3.1
	14 – 15	191	498	135.7 ± 0.7	236.4 ± 2.6
	15 – 16	190	500	121.1 ± 0.7	212.9 ± 2.5
	16 – 17	191	503	110.5 ± 0.6	194.5 ± 2.2
	17 – 18	191	500	98.9 ± 0.6	173.1 ± 2.0
	18 – 19	192	502	85.6 ± 0.5	149.6 ± 1.7
	19 – 20	189	500	73.1 ± 0.5	129.3 ± 1.6
	20 – 21	191	502	66.5 ± 0.4	116.8 ± 1.4
	21 – 22	192	502	58.8 ± 0.5	102.7 ± 1.3
	22 – 23	191	501	50.53 ± 0.32	88.6 ± 1.0
	23 – 24	194	501	42.3 ± 0.5	73.0 ± 1.1
	24 – 25	190	501	36.88 ± 0.27	65.0 ± 0.8
	25 – 26	190	502	34.04 ± 0.34	60.1 ± 0.9
	26 – 27	191	501	29.97 ± 0.19	52.5 ± 0.6
	27 – 28	194	500	28.10 ± 0.29	48.4 ± 0.7
	28 – 29	191	497	27.76 ± 0.22	48.3 ± 0.6
	29 – 30	192	501	27.66 ± 0.19	48.2 ± 0.6
	30 – 31	191	500	26.82 ± 0.18	46.9 ± 0.6
	31 – 32	189	501	25.00 ± 0.18	44.3 ± 0.5
	32 – 33	192	499	22.29 ± 0.18	38.7 ± 0.5
	33 – 34	192	501	18.12 ± 0.15	31.6 ± 0.4
	34 – 35	190	499	14.20 ± 0.12	24.9 ± 0.3
	35 – 36	55	447	3.66 ± 0.06	19.9 ± 0.4
	37 – 38	52	445	2.27 ± 0.05	13.0 ± 0.3
	39 – 40	53	449	1.63 ± 0.03	9.2 ± 0.2
	40 – 42	64	441	1.77 ± 0.04	8.1 ± 0.2
	42 – 44	59	454	1.16 ± 0.03	6.0 ± 0.2
	46 – 48	49	449	0.75 ± 0.02	4.6 ± 0.2
	50 – 52	49	450	0.60 ± 0.02	3.7 ± 0.1

	Depth<br>mm	Crust mass<br>mg	^{9}Be carrier<br>μg	^{10}Be/^{9}Be<br>10^{-13} at/at	^{10}Be<br>10^{7} at/g
(b)	00 – 01	38	607	223.0 ± 0.8	2381 ± 25
	01 – 02	55	607	315.1 ± 0.9	2335 ± 24
	02 – 03	202	503	1182.6 ± 2.0	1968 ± 20
	03 – 04	202	504	870.4 ± 1.7	1451 ± 15
	04 – 05	202	505	643.5 ± 1.4	1075 ± 11
	05 – 06	201	506	499.1 ± 1.3	840 ± 9
	06 – 07	201	506	406.3 ± 1.2	683 ± 7
	07 – 08	202	504	337.7 ± 1.0	563 ± 6
	08 – 09	202	505	282.0 ± 0.9	471 ± 5
	10 – 11	193	501	189.3 ± 0.8	328.4 ± 3.5
	12 – 13	192	504	124.6 ± 0.7	218.5 ± 2.5
	13 – 14	55	609	25.05 ± 0.21	184.9 ± 2.4
	14 – 15	193	505	90.6 ± 0.5	158.4 ± 1.8
	16 – 17	191	504	62.5 ± 0.4	110.2 ± 1.3
	18 – 19	192	506	47.05 ± 0.32	82.9 ± 1.0
	20 – 21	191	505	34.71 ± 0.25	61.3 ± 0.8
	22 – 23	190	508	27.23 ± 0.20	48.6 ± 0.6
	24 – 25	51	447	7.38 ± 0.09	43.2 ± 0.7
	26 – 27	52	451	6.07 ± 0.08	35.2 ± 0.6
	28 – 29	56	451	5.15 ± 0.07	27.7 ± 0.5
	30 – 31	59	450	3.00 ± 0.05	15.3 ± 0.3
	32 – 33	53	451	1.90 ± 0.04	10.8 ± 0.2
	34 – 35	52	449	1.21 ± 0.03	7.0 ± 0.2
	36 – 37	53	453	0.94 ± 0.02	5.4 ± 0.1
	38 – 39	50	451	0.66 ± 0.02	4.0 ± 0.1
	41 – 43	53	451	0.54 ± 0.02	3.1 ± 0.1
	45 – 47	57	446	0.35 ± 0.01	1.8 ± 0.1
	49 – 51	50	450	0.24 ± 0.01	1.5 ± 0.1

	Depth mm	Crust mass mg	^{9}Be carrier μg	^{10}Be/^{9}Be 10^{-13} at/at	^{10}Be 10^{7} at/g
4DR	00 – 02	58	443	393 ± 8	2001 ± 46
	00 – 03	35	446	170 ± 4	1456 ± 34
	04 – 05	38	447	73.1 ± 1.6	580 ± 14
	05 – 06	35	446	54.6 ± 1.2	458 ± 11
	06 – 07	40	442	45.6 ± 1.0	334 ± 8
	07 – 08	32	454	29.5 ± 0.6	278 ± 7
	08 – 09	38	451	28.3 ± 0.6	222 ± 5
	09 – 10	41	450	24.1 ± 0.5	177 ± 4
	10 – 11	32	449	15.78 ± 0.35	149 ± 4
	11 – 12	38	454	14.72 ± 0.33	117.5 ± 2.9
	12 – 13	35	457	10.74 ± 0.25	93.7 ± 2.4
	13 – 14	36	452	8.49 ± 0.20	72.0 ± 1.8
	14 – 15	38	453	6.65 ± 0.16	53.6 ± 1.4
	15 – 16	40	456	6.29 ± 0.16	48.3 ± 1.3
	16 – 17	38	452	5.27 ± 0.13	41.9 ± 1.1
	17 – 18	41	446	5.72 ± 0.14	41.4 ± 1.1
	18 – 19	43	454	3.71 ± 0.10	26.1 ± 0.8
	19 – 20	42	458	2.94 ± 0.09	21.6 ± 0.7
	20 – 21	44	455	2.90 ± 0.09	20.0 ± 0.7
	21 – 22	38	450	2.40 ± 0.08	18.9 ± 0.7
	22 – 23	36	454	1.98 ± 0.08	16.7 ± 0.7
	23 – 24	38	454	1.82 ± 0.07	14.4 ± 0.6
	24 – 25	35	456	1.47 ± 0.06	13.0 ± 0.6

Supplementary Table 1 Measured ^{10}Be depth profile in drill-holes (a) and (b) of the ferromanganese crust VA13/2-237KD and the drill-hole of the ferromanganese crust SO142-4DR. Crust weighing and carrier addition introduces a statistical uncertainty of 0.5% each and the statistical uncertainty of the standard measurement was 0.7% which were added to the final results. The systematic uncertainty of the used standard SMD-Be-12 ([4]) is 1.8% and of the carrier solution is 0.5%, which only need to be considered for the absolute values but not for the depth profile. The measured ^{10}Be/^{9}Be ratio from AMS can be converted to a ^{10}Be concentration by accounting for carrier addition. The ^{9}Be concentration from ICP-MS could be used to calculate an intrinsic authigenic ^{10}Be/^{9}Be ratio.

References

[1] Koll, D. *et al.* Element Separation Chemistry and Cosmogenic ^{10}Be-Dating of a Ferromanganese Crust. *Nuclear Instruments and Methods in Physics Research Section B: Beam Interactions with Materials and Atoms* **530**, 53–58 (2022). URL https://www.sciencedirect.com/science/article/pii/S0168583X22002221.

[2] Halbach, P. E., Jahn, A. & Cherkashov, G. *Marine Co-Rich Ferromanganese Crust Deposits: Description and Formation, Occurrences and Distribution, Estimated World-wide Resources*, 65–141 (Springer International Publishing, 2017). URL https://doi.org/10.1007/978-3-319-52557-0_3.

[3] Koschinsky, A. & Halbach, P. E. Sequential leaching of marine ferromanganese precipitates: Genetic implications. *Geochimica et Cosmochimica Acta* **59**, 5113–5132 (1995). URL https://www.sciencedirect.com/science/article/pii/0016703795003584.

[4] Akhmadaliev, S., Heller, R., Hanf, D., Rugel, G. & Merchel, S. The new 6 MV AMS-facility DREAMS at Dresden. *Nuclear Instruments and Methods in Physics Research Section B: Beam Interactions with Materials and Atoms* **294**, 5–10 (2013). URL https://www.sciencedirect.com/science/article/pii/S0168583X12000961.
